# Intratumoral heterogeneity of the therapeutical response to gemcitabine and metformin

**DOI:** 10.18632/oncotarget.10892

**Published:** 2016-07-28

**Authors:** Dietmar Zechner, Florian Bürtin, Ann-Christin Albert, Xianbin Zhang, Simone Kumstel, Maria Schönrogge, Josefine Graffunder, Hao-Yu Shih, Sarah Müller, Tobias Radecke, Robert Jaster, Brigitte Vollmar

**Affiliations:** ^1^ Institute for Experimental Surgery, Rostock University Medical Center, 18057 Rostock, Germany; ^2^ Division of Gastroenterology, Department of Medicine II, Rostock University Medical Center, 18057 Rostock, Germany

**Keywords:** pancreatic adenocarcinoma, chemotherapy, syngeneic orthotopic cancer model, microenvironment, pH Blot

## Abstract

Cancer heterogeneity and microenvironmental aspects within a tumor are considered key factors influencing resistance of carcinoma cells to distinct chemotherapeutical agents. We evaluated a high concentration of metformin in combination with gemcitabine on a syngeneic orthotopic mouse model using 6606PDA cells. We observed reduced tumor size and reduced cancer cell proliferation after three weeks of chemotherapy with either compound and noticed an additive effect between gemcitabine and metformin on tumor weight. Interestingly, distinct areas of the carcinoma responded differently to either compound. Metformin inhibited the proliferation of cancer cells close to the desmoplastic reaction, whereas gemcitabine inhibited the proliferation of cancer cells mainly 360–570 μm distant to the desmoplastic reaction. Indeed, co-culture of pancreatic stellate cells with 6606PDA, 7265PDA or MIA PaCa-2 cells increased gemcitabine resistance. Metformin resistance, however, was increased by high glucose concentration in the medium. Other factors such as hypoxia or the pH of the medium had no influence on gemcitabine or metformin induced inhibition of cancer cell proliferation. These data demonstrate a spatial heterogeneity in drug resistance within pancreatic adenocarcinomas and that microenvironmental aspects such as supply of glucose and the presence of pancreatic stellate cells regulate cancer cell sensitivity towards metformin or gemcitabine.

## INTRODUCTION

Although multiple chemotherapies for the treatment of pancreatic cancer have been evaluated, the 5-year survival rate of pancreatic cancer patients is still only 7% [1]. For adjuvant therapy chemotherapeutic regiments such as gemcitabine or 5-Fluoruracil with follinic acid are recommended [2]. For the treatment of advanced or metastatic pancreatic cancer gemcitabine in combination with nab-paclitaxel or a combination known as FOLFIRINOX (5-fluoruracil, leucovorin, oxaliplatin, irinotecan) can be used [3, 4]. Since all chemotherapies offer modest mortality benefit, novel combinatorial chemotherapies continue to be tested [5, 6]. Lately the benefit of metformin, a traditional diabetes type II medication, has been evaluated in clinical studies. These studies demonstrated that metformin at a low dose typical for glycemic control is unlikely to benefit patients with pancreatic cancer when used in combination with traditional cytostatic agents [7, 8]. However a subgroup of patients with high metformin concentration (> 1 mg/L) in the blood seemed to have an improved survival [7].

The efficacy of chemotherapies is often jeopardized by the heterogeneity of carcinomas [9, 10]. Heterogeneity may exist between carcinomas of different patients, but also within a single carcinoma [11]. This intratumoral heterogeneity can be caused by cell intrinsic mechanisms such as the accumulation of distinct sets of mutations in distinct subpopulations of cancer cells [12]. Another prominent cause for intratumoral heterogeneity is the influence of the microenvironment on cancer cells [10]. For example, certain aspects of the microenvironment such as the presence of a tumor stroma can restrict the ability of chemotherapeutic agents to reach the carcinoma cells and cause thereby drug resistance [13–15]. These observations already lead to the development of experimental drugs, with the goal to reduce the tumor stroma [13–15]. However, the benefit of disrupting the tumor stroma is highly controversial [13–17]. Understanding the interaction of cancer heterogeneity, microenvironmental aspects and drug resistance will continue to be important to develop novel therapies [9, 10, 18].

In this study we treated pancreatic adenocarcinomas with gemcitabine and a high metformin dose and observed local heterogeneity in the inhibition of cancer cell proliferation in response to each drug. Based on these *in vivo* observations, we characterized microenvironmental aspects, such as glucose supply, pH, hypoxia and the presence of pancreatic stellate cells on drug resistance of pancreatic cancer cells.

## RESULTS

### Gemcitabine and metformin reduce cell proliferation in distinct carcinoma regions

**Figure 1:**
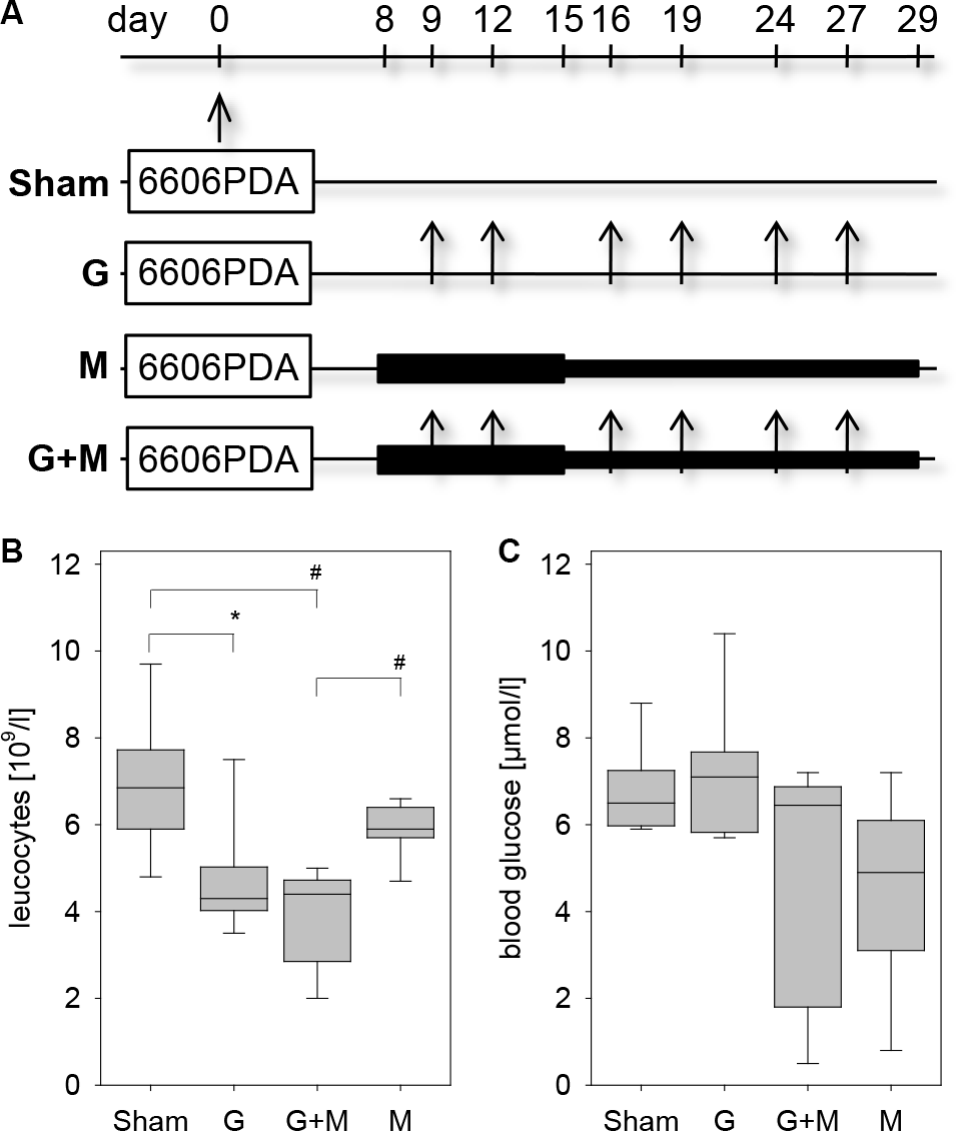
Characterization of the pancreatic cancer model (**A**) 6606PDA cells were injected on day 0 into the murine pancreas. Control cohorts (Sham) were ip injected with an appropriate vehicle, whereas other cohorts were ip injected twice a week with gemcitabine (G), daily with metformin (M) or with gemcitabine plus metformin (G + M). (**B**) Treatment with gemcitabine or gemcitabine and metformin reduced the concentration of leucocytes in the blood on day 29. (**C**) Administration of metformin or gemcitabine plus metformin modestly reduced the blood glucose concentration as quantified on day 14. Significant differences: *p = 0.005, ^#^*p* < 0.001.

**Figure 2:**
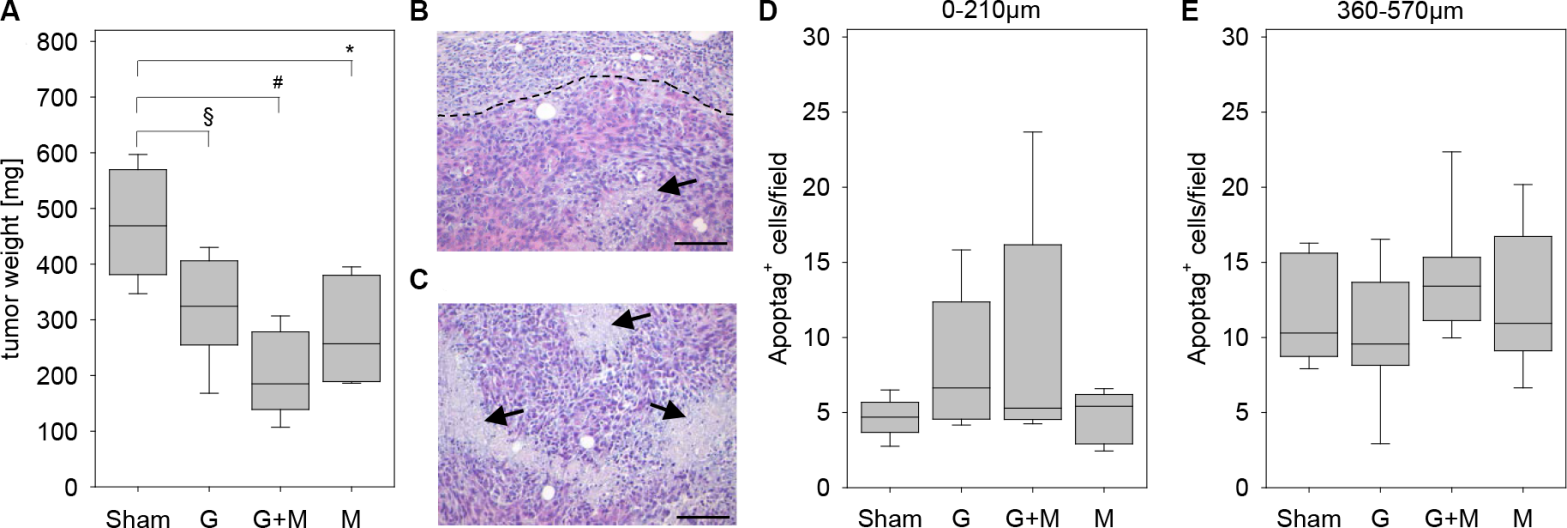
Quantification of tumor weight and cell death (**A**) Quantification of the tumor weight of the indicated mouse cohorts on day 29. (**B**) The histological image of a tumor illustrates a necrotic area (arrow) close to the border (broken line) between carcinoma and desmoplastic reaction. (**C**) The histological image of a tumor presents several necrotic areas (arrows) inside the carcinoma. Negligible differences in the quantification of cell death between the indicated mouse cohorts as quantified at 0–210 μm distance (**D**) or at 360–570 μm distance (**E**) to the desmoplastic reaction. Significant differences: *p = 0.004, ^#^*p* < 0.001. Tendentious difference: ^§^*p* = 0,015. Bar = 100 μm.

In order to evaluate the effect of metformin in combination with gemcitabine on the pathophysiology of pancreatic cancer *in vivo*, we injected 6606PDA cells into the pancreas of mice. Distinct mouse cohorts were sham treated or injected intraperitoneally with gemcitabine, metformin or both substances for three weeks (Figure 1A). Gemcitabine application was effective, since it reduced the number of leucocytes (Figure 1B). The application of metformin resulted in a nonsignificant reduction in the concentration of blood glucose (Figure 1C). After three weeks of gemcitabine as well as metformin application reduced tumor weight was observed when compared to sham treated mice (Figure 2A). Treating mice with both substances lead to an even larger reduction of the tumor weight (Figure 2A). Independent of treatment, all tumors had similar histological features as described previously [19, 20]. They were cyst-like, completely encapsulated by fibroblast like cells and had few necrotic areas close to the encapsulation (0–120 μm distance from the desmoplastic reaction), but more necrotic areas at 360–570 μm distance from the desmoplastic reaction (Figure 2B and 2C). However, major differences in cell death between distinct cohorts of mice were neither observed in close proximity to the desmoplastic reaction nor at 360–570 μm distance to the tumor stroma (Figure 2D and 2E).

**Figure 3:**
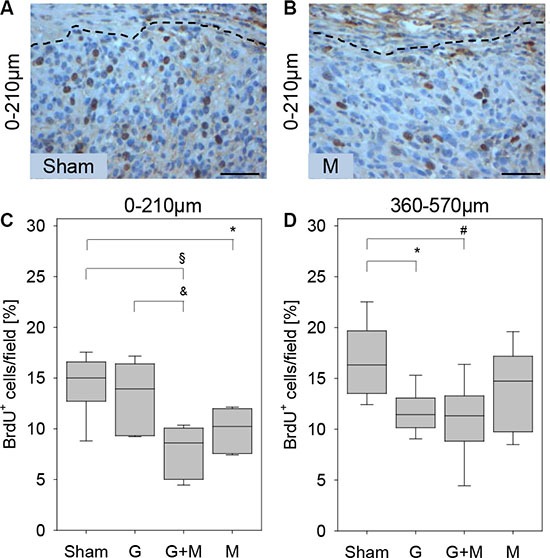
Quantification of proliferation *in vivo* The histological images of a tumor illustrate that in 0–210 μm distance to the desmoplastic reaction more BrdU^+^ cells are observed in sham (Sham) treated mice (**A**) when compared to metformin (M) treated mice (**B**). (**C**) Metformin (M) or gemcitabine plus metformin (G + M) treatment reduces the number of BrdU^+^ cells (quantified at 0–210 μm distance to the desmoplastic reaction). (**D**) Gemcitabine (G) or gemcitabine plus metformin (G + M) treatment reduces the number of BrdU^+^ cells (quantified at 360–570 μm distance to the desmoplastic reaction). Significant differences: *p = 0.006, ^§^*p* < 0.002, ^&^*p* < 0.009 in panel C and *p = 0.004 in panel D. Tendentious difference: ^#^*p* = 0,015 in panel D. Bar = 50 μm.

Interestingly, inhibition of proliferation by gemcitabine and metformin was dependent on the distance to the desmoplastic reaction (Figure 3). When evaluating the carcinomas 0–210 μm close to the desmoplastic reaction metformin as well as gemcitabine plus metformin treatment reduced the number of proliferating cancer cells significantly, whereas gemcitabine treatment had only a moderate effect on cancer cell proliferation (Figure 3A–3C). When evaluating the carcinomas 360–570 μm from the desmoplastic reaction gemcitabine as well as gemcitabine plus metformin treatment reduced the number of proliferating cancer cells significantly, whereas metformin treatment had only a moderate effect on cancer cell proliferation (Figure 3D). Thus, local differences in the proliferation rate of carcinoma cells, in response to gemcitabine and metformin treatment, can be observed *in vivo*.

### Expression of gemcitabine and metformin transport proteins

We also evaluated the expression of proteins involved in the transport of gemcitabine or metformin. 6606PDA cells expressed metformin transporter proteins such as the organic cation transporter OCT1/2 with a theoretical molecular weight of 62 kDa (Supplementary Figure 1A). The expression of plasma membrane monoamine transporter (PMAT) with a theoretical molecular weight of 58 kDa was detected in the intestine, but in 6606PDA cells two proteins with an apparent molecular weight of 70 kDa and 55 kDa were observed (Supplementary Figure 1B). Possibly these proteins might be splice variants or glycosylated forms of PMAT. OCT1/2 as well as PMAT was detected throughout 6606PDA derived carcinomas *in vivo* (Supplementary Figure 1C and 1D). This suggests that the observed metformin induced reduction of cancer cell proliferation primarily 0–210 μm close to the desmoplastic reaction cannot be explained by the expression of these two transport proteins.

**Figure 4:**
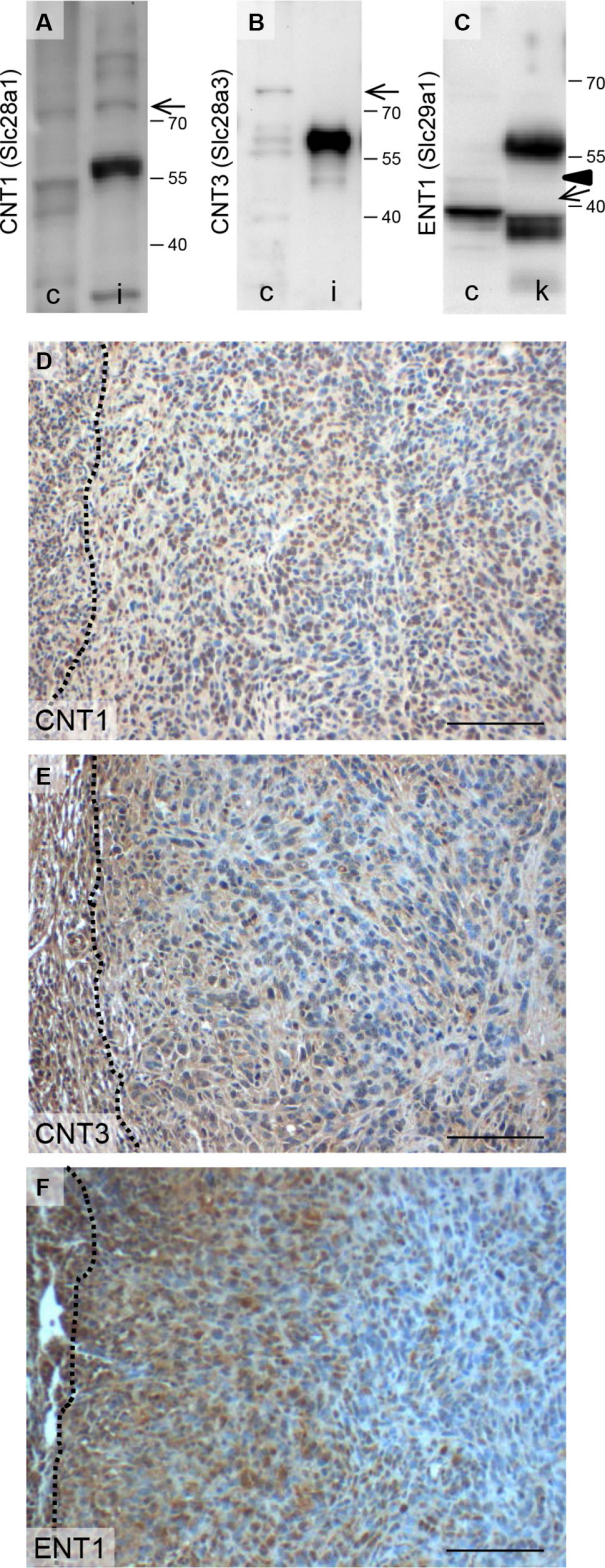
Expression of proteins involved in gemcitabine transport Analysis of CNT1 (**A**), CNT3 (**B**), ENT1 (**C**) expression in 6606PDA cells (c), kidney (k) and intestine (i) by Western Blotting (the arrows point at CNT1, CNT3 or a known functional isoform of Ent-1, called mENT1D11 [21] with a theoretical molecular weight of 71 kDa, 78 kDa or 39 kDa, respectively (Arrowhead points at full length ENT1 with a theoretical molecular weight of 50 kDa). Evaluation of CNT1 (**D**), CNT3 (**E**) or Ent1 (**F**) expression in carcinomas by immunohistochemistry (the broken lines indicate the border between the carcinoma and the desmoplastic reaction). Bar = 100 μm.

In addition, we evaluated the expression of known gemcitabine transport proteins. We observed that nucleoside import proteins such as the concentrative nucleoside transporters CNT1 with a theoretical molecular weight of 71 kDa as well as CNT3 with a theoretical molecular weight of 78 kDa were expressed by 6606PDA cells (Figure 4A and 4B). The full length of the equilibrative nucleoside transporter 1 (ENT1) with a theoretical molecular weight of 50 kDa was very moderately expressed in 6606PDA cells (Figure 4C). A known functional isoform, however, called mENT1Δ11 [21] with a theoretical molecular weight of 39 kDa, was highly expressed in this cell line (Figure 4C). CNT1 as well as CNT3 was expressed throughout the carcinomas *in vivo* (Figure 4D and 4E). ENT1, however, was mainly expressed close to the desmoplastic reaction (Figure 4F). Since ENT1 is implicated in re-exporting gemcitabine from cells [22], ENT1 expression close to the desmoplastic reaction might reduce gemcitabine induced inhibition of cell proliferation in this area of the tumor. Thus, the expression pattern of gemcitabine, but not of metformin transporter proteins might explain the observed local differences in cell proliferation in response to these two therapies.

### Microenvironmental aspects regulating gemcitabine resistance *in vitro*

**Figure 5:**
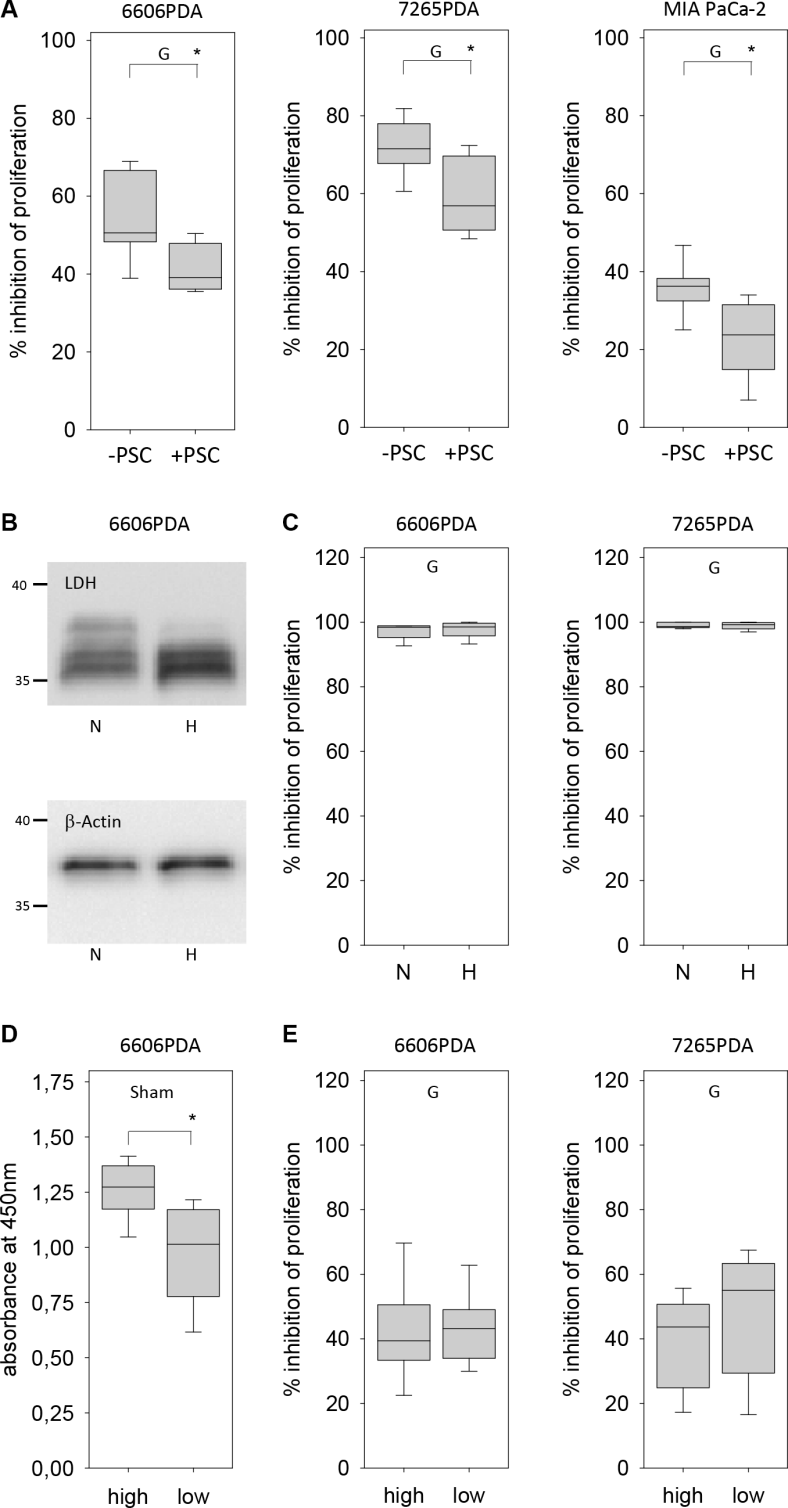
Regulation of gemcitabine resistance of carcinoma cells by pancreatic stellate cells, hypoxia and glucose (**A**) Gemcitabine (G) induced inhibition of cancer cell proliferation is reduced by co-culturing 6606PDA, 7265PDA or MIA PaCa-2 cells with PSCs. (**B**) Analysis of lactate dehydrogenase A (LDH) and β-actin expression in 6606PDA cells cultured under normoxic (N) and hypoxic (H) conditions. (**C**) No difference in gemcitabine (G) induced inhibition of 6606PDA or 7265PDA proliferation under normoxic (N) or hypoxic (H) conditions. (**D**) Untreated (Sham) 6606PDA cells proliferate more in high glucose medium when compared to low glucose medium. (**E**) Glucose concentration does not influence gemcitabine (G) induced inhibition of 6606PDA or 7265PDA cell proliferation. Significant difference: *p ≤ 0.017 in panel B, *p = 0.006 in panel D.

To explore the option if tumor stroma cells might influence the resistance of pancreatic cancer cells towards gemcitabine, we co-cultured pancreatic stellate cells (PSC) with 6606PDA cells. PSCs were treated with mitomycine C in order to inhibit their proliferation. A co-culture of 6606PDA cells and mitomycine C treated PSCs resulted in modest increase of the proliferation rate when compared to 6606PDA cells (6606PDA: 1.786/1.371–2.013 6606PDA + PSC: 1.880/1.650–2.54 PSC: 0.021/.0.19–0.039 median/interquartile range in absorption at 450 nm). However, gemcitabine administration to 6606PDA cells co-cultured with PSCs resulted in a significantly weaker inhibition of cell proliferation when compared to 6606PDA cell, which were grown without PSCs (Figure 5A). We also evaluated other pancreatic cancer cell lines. Gemcitabine administration to murine 7265PDA and human MIA PaCa-2 cells co-cultured with PSCs resulted also in a significantly weaker inhibition of cell proliferation when compared to carcinoma cells, which were grown without PSCs (Figure 5A). We also investigated if hypoxia influences the resistance of pancreatic cancer cells towards gemcitabine. Hypoxia induced the expression of lactate dehydrogenase A, as described in other cell lines [23], but failed to have any influence on gemcitabine induced inhibition of cancer cell proliferation, when evaluating 6606PDA or 7265PDA cells (Figure 5B and 5C). When studying MIA PaCa-2 cells we also did not observe a major influence of oxygen concentration on gemcitabine induced inhibition of proliferation (Normoxia: 78.8/74.6–85.1; hypoxia: 76.9/68.2–82.6 median/interquartile range in % inhibition). However, all three cell lines modestly increased their proliferation under hypoxic conditions (6606PDA: normoxia 0.95/0.87–1.19, hypoxia 1.14/1.00–1.36; 7265PDA: normoxia 1.10/0.98–1.14, hypoxia 1.16/1.09–1.22; MIA PaCa-2: normoxia 0.78/0.67–0.88, hypoxia 0.94/0.80–1.12 median/interquartile range in absorption at 450 nm). In addition, we explored, if glucose concentration in the medium influences the resistance of pancreatic cancer cells towards gemcitabine. High glucose concentration increased the proliferation of untreated 6606PDA cells (Figure 5D). However, glucose concentration had no major influence on gemcitabine induced inhibition of cancer cell proliferation of 6606PDA or 7265PDA cells (Figure 5E). Glucose concentration had also no major influence on gemcitabine induced inhibition of cancer cell proliferation of MIA PaCa-2 cells (high glucose: 30.7/13.3–37.7, low glucose: 36.2/24.0–45.1 median/interquartile range in % inhibition of proliferation).

**Figure 6:**
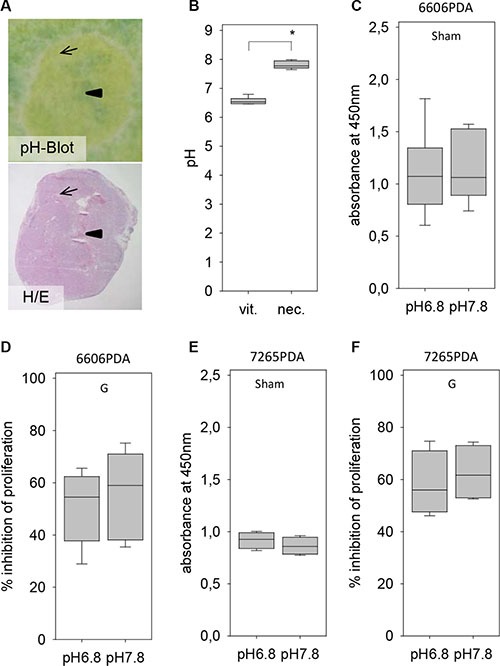
Evaluation of the pH within the tumor and its influence on gemcitabine resistance (**A**) Visualization of the pH within a tumor by pH Blot (arrow: vital carcinoma, arrowhead: central necrosis) and comparison to a hematoxylin/eosin (H/E) stained tumor section. (**B**) Quantification of the pH in vital tumor tissue (vit.) and the necrotic central area (nec.). (C) No difference in the proliferation rate of untreated (Sham) 6606PDA cells grown at pH 6.8 or pH 7.8. (**D**) Gemcitabine (G) induced inhibition of proliferation is also not influence by the pH of the medium. (**E**) No difference in the proliferation rate of untreated (Sham) 7265PDA cells grown at pH 6.8 or pH 7.8. (**F**) Gemcitabine (G) induced inhibition of 7265 PDA proliferation is also not influenced by the pH of the medium. Significant difference: *p = 0.002.

Another microenvironmental aspect, which could be different between distinct areas of the carcinoma, was the pH of the tissue. Indeed, blotting cryo-sectioned carcinomas on a pH-paper revealed a higher pH in the inside of the tumor, which was characterized by an extensive central necrosis, when compared to vital tissue close to the rim of the carcinoma (Figure 6A). The average pH in the central necrosis was 7.8 whereas the pH in the vital tissue on the outside of the tumor was significantly lower (Figure 6B). We evaluated, if the pH of the medium influences the resistance of pancreatic cancer cells towards gemcitabine *in vitro*. A ten-fold difference in the H^+^ ion concentration in the medium had remarkable little influence on the proliferation of untreated 6606PDA cells (Figure 6C) or on gemcitabine induced inhibition of cancer cell proliferation (Figure 6D). Differences in the H^+^ ion concentration in the medium had also little influence on the proliferation of untreated 7265PDA cells (Figure 6E) or on gemcitabine induced inhibition of cancer cell proliferation (Figure 6F). No major influence of the pH value on proliferation was also noticed when studying MIA PaCa-2 cells (data not shown).

Thus, sensitivity of 6606PDA, 7265PDA or MIA PaCa-2 cells towards gemcitabine is not influenced by glucose concentration or H^+^ ion concentration in the medium. It is also not influenced by hypoxia, but is reduced by co-culture with pancreatic stellate cells.

### Microenvironmental aspects regulating metformin resistance *in vitro*

**Figure 7:**
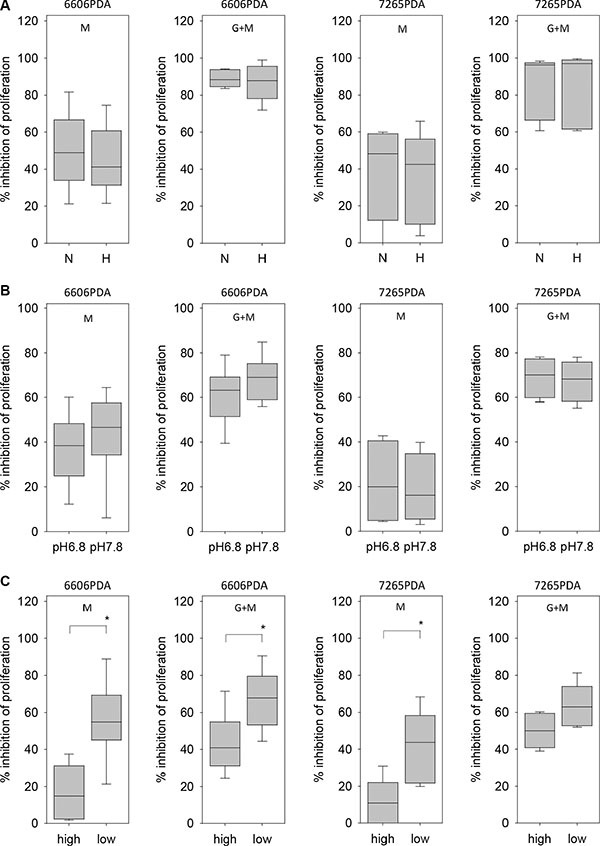
Regulation of metformin resistance of 6606PDA cells by hypoxia, pH and glucose (**A**) No difference in metformin (M) or gemcitabine plus metformin (G + M) induced inhibition of 6606PDA or 7265PDA proliferation under normoxic (N) or hypoxic (H) conditions. (**B**) No major difference in metformin (M) or gemcitabine plus metformin (G + M) induced inhibition of 6606PDA or 7265PDA proliferation when cells were grown in medium with pH 6.8 or pH 7.8. (**C**) Increased inhibition of 6606PDA or 7265PDA proliferation by metformin (M) or gemcitabine plus metformin (G + M) in low glucose medium when compared to high glucose medium. Significant differences: *p ≤ 0.032 (M), *p = 0.008 (G + M).

We also explored which microenvironmental aspects can influence the resistance of pancreatic cancer cells towards metformin. Hypoxia failed to have a significant influence on metformin or metformin plus gemcitabine induced inhibition of 6606PDA, 7265PDA or MIA PaCa-2 cell proliferation (Figure 7A and data not shown). Moreover, a ten-fold difference in the H^+^ ion concentration in the medium had also not a major influence on metformin or metformin plus gemcitabine induced inhibition of cancer cell proliferation (Figure 7B and data not shown). We also explored, if glucose concentration in the medium influences the resistance of pancreatic cancer cells towards metformin or gemcitabine plus metformin treatment. Proliferation of 6606PDA cells grown in medium with low glucose concentration were significantly stronger inhibited by metformin or gemcitabine plus metformin treatment than cells grown in high glucose concentration (Figure 7C). A stronger inhibition of proliferation by metformin or gemcitabine plus metformin treatment in medium of low glucose concentration was also observed with 7265PDA cells (Figure 7C). However, this result was not confirmed when evaluating MIA PaCa-2 cells (metformin: high glucose 34.4/24.6–36.5, low glucose 11.8/3.2–24.6; metformin plus gemcitabine: high glucose 47.6/28.0–58.2, low glucose 43.5/28.7–52.1 median/interquartile range in % inhibition).

Thus, resistance of 6606PDA or 7265PDA cells towards metformin is not influenced by H^+^ ion concentration in the medium or by hypoxia, but is decreased by low glucose concentration in the medium.

## DISCUSSION

The presented *in vivo* data demonstrate that gemcitabine inhibits cancer cell proliferation primarily in cancer cells that are situated distant to the desmoplastic reaction in a more central position of cyst like carcinomas (Figure 3). Usually the function of the desmoplastic reaction is described as barrier, which blocks access of gemcitabine to cancer cells and thereby causes resistance to this drug [13–15]. However, the observed spatial heterogeneity in response to gemcitabine in the carcinomas of the presented mouse model cannot be explained by this hypothesis, since carcinoma regions in the inside of the tumors, which should even be better shielded from gemcitabine, are most sensitive to gemcitabine (Figure 3). Our data rather suggest a more active role of the desmoplastic reaction in conferring gemcitabine resistance to carcinoma cells. This hypothesis is supported by *in vitro* data demonstrating that co-culturing pancreatic stellate cells with 6606PDA cells or other pancreatic cancer cell lines reduces gemcitabine induced inhibition of cancer cell proliferation, although cancer cells were in free contact to gemcitabine supplemented medium (Figure 5A–5C).

While gemcitabine is well established as a therapeutical agent for the treatment of pancreatic adenocarcinoma, the benefit of metformin is highly disputed. Many experimental studies in mice used a high dose of metformin, such as 125 mg/kg per day [24], 150 mg/kg per day [25] or 250 mg/kg per day [26] and observed reduced growth of pancreatic cancer. Clinical studies, however, demonstrated that metformin at a low dose typical for glycemic control (up to 2 g/patient per day) is unlikely to benefit patients with pancreatic cancer [7, 8]. However a subgroup of patients with high metformin concentration in the blood seemed to have an improved survival [7]. In our study we used a high dose of 250 mg/kg metformin per day for the first week and observed in 25% of gemcitabine plus metformin treated mice and 43% of metformin treated mice a strong reduction of the blood glucose concentration (0.5 to 3.9 mM) on day 14. We therefore reduced the dose of metformin in the following two weeks to 125 mg/kg per day. This resulted in a recovery of the blood glucose concentration in these mice. This suggests that a daily dose of 125 mg/kg metformin can be administered safely in C57Bl/6J mice, but that the administration of 250 mg/kg can have adverse effects (at least when administered in form of ip injections). Considering that in multiple preclinical studies more than 50 mg/kg metformin very consistently reduced tumor weight [24–28], it might be worth pursuing clinical studies with a higher dose of metformin.

So far no definite mechanism has been identified how metformin inhibits cancer cell proliferation. One favored option is that metformin inhibits oxidative phosphorylation in mitochondria, which leads to an energy crisis within cells [29]. In response to this energy stress adaptive responses of the cell reduces energy consuming processes, such as proliferation [30]. We observed, however, that hypoxia, which usually also causes an energy crisis in cells, did not have a significant influence on the inhibition of 6606PDA, 7265PDA or MIA PaCa-2 cell proliferation by metformin (Figure 7A and data not shown). These data are consistent with the hypothesis that many carcinoma cells produce energy predominantly via enhanced glycolysis even under aerobic conditions [31]. This Warburg effect might also occur in 6606PDA cells, since we observed that this cell line produced only about 18% less lactate under normoxic conditions when compared to hypoxic conditions (data not shown). Nevertheless, these considerations raise the question how metformin, a well characterized inhibitor of the respiratory chain complex I in mitochondria [32, 33], inhibits cell proliferation independent of oxygene supply. Either one has to assume that 1% oxygene is sufficient for some oxidative phosphorylation to occur and its inhibition by metformin reduces cell proliferation, or metformin inhibits cancer cell proliferation by modulating key processes completely distinct to the inhibition of the respiratory chain such as inhibition of hexokinase or inhibition of gylcerophosphate dehydrogenase [34, 35].

Interestingly, the presented *in vivo* data demonstrate that metformin inhibits cancer cell proliferation in a spatially distinct area of the carcinoma when compared to gemcitabine (Figure 3). This spatial heterogeneity in the response to two distinct drugs causes an additive effect on tumor growth (Figures 2A and 3). Combinatorial therapies have been defined to work in two distinct ways: i) one agent reinforces the action of another agent, or ii) two drugs may combine to exert effects that are distinct from either individual compound [36]. These classical assumptions how combinatorial therapies work are based on the idea that distinct drugs inhibit identical cancer cells by different mechanisms. The presented data, however, demonstrate that distinct agents might also have a combinatorial effect on the tumor by targeting distinct areas of a carcinoma. Pursuing combinatorial therapies to target distinct subpopulations of a carcinoma might be a promising option for the future. However, appropriate animal models must be developed for this purpose.

## MATERIALS AND METHODS

### Cell culture

The murine cell line, 6606PDA and 7265PDA were a gift of Prof. Tuveson (University of Cambridge, UK) and were grown in DMEM high glucose medium (Biochrom GmbH, Berlin, Germany) as previously described [19, 20]. The human MIA PaCa-2 cells were ordered from ATCC (LGC Standards GmbH, Wesel, Germany). The PSCs were isolated from the pancreas of C57BL/6J mice by collagenase digestion of the organ and by Nycodenz density gradient centrifugation as previously described [37]. These cells were expanded in Iscove's-medium (Biochrom GmbH, Berlin, Germany) supplemented with 17% fetal calf serum (FCS), 1% non-essential amino acids, 100 U/ml penicillin and 100 μg/ml streptomycin for one to two weeks. All experiments were performed with passaging the cells no more than 2 times.

### Evaluation of proliferation *in vitro*

For evaluating cell proliferation all cells were seeded in a 96 well plate and grown in either DMEM high glucose medium (Biochrom GmbH) or as indicated in Figures 5D, 5E and 7C in low glucose medium (Dulbeccos MEM from Biochrom after adding 0,5 g/L glucose). Both media were supplemented with 10% fetal calf serum. For co-culture experiments of PSCs with 6606PDA, 7265PDA or MIA PaCa-2 cells (Figure 5A), the proliferation of PSCs was stopped by treating them with 10 μg/ml mitomycine D (Sigma-Aldrich, St. Louis, USA) for 2 hours, followed by washing the cells twice with PBS. For some experiments (Figure 6C–6F and Figure 7B) DMEM high glucose medium (Biochrom GmbH) was adjusted to pH 6.8 or pH 7.8 with 1 M HCl or 1 M NaOH. The media were retitrated until the pH remained stable after equilibrating the medium in a tissue culture incubator for 24 hours. In order to mimic lactate production in the carcinoma, the medium with pH 6.8 was also supplemented with 10 mM lactate (Sigma-Aldrich). For growing cells under hypoxic conditions, the cell culture dishes were placed 24 hours after seeding the cells in an Innova CO-48 incubator (New Brunswick Scientific Co, Edison Edison, USA) under 1% oxygene supply. In all experiments 6606PDA and 7265PDA cells were treated 24 hours after seeding with control media, 100 nM gemcitabine, 20 mM metformin or 100 nM gemcitabine plus 20 mM metformin. MIA PaCa-2 cells were treated with control media, 25 nM gemcitabine, 20 mM metformin or 25 nM gemcitabine plus 20 mM metformin. The cells were treated with these agents for 24 hours (glucose experiments: Figures 5D and 7C; PSC experiments: Figure 5A, pH experiments: Figure 6C–6F and Figure 7B) or 72 hours (hypoxia experiments: Figure 5C and 7A) and the BrdU labeling reagent (Roche Diagnostics, Mannheim, Germany) was added within the last 24 hours. The incorporation of BrdU was then quantified with the colorimetric Cell Proliferation ELISA (Roche Diagnostics).

### Western blots

Western blots were performed by separating cell lysate on SDS polyacryl gels and transferring the proteins to a polyvinyldifluoride membrane (Immobilon-P; Millipore, Eschborn, Germany) as described previously [19]. The membranes were blocked with 2.5% (wt/vol.) BSA or 5% (wt/vol.) milk powder (only for the analysis of OCT1/2) and incubated overnight at 4°C with a goat anti-CNT1 (Santa Cruz Biotechnology, Santa Cruz, USA, code sc48457, dilution: 200×), rabbit anti-CNT3 (Santa Cruz Biotechnology, code sc134529, dilution: 1000×), rabbit anti-ENT1 (Abcam, Cambridge, UK, code ab135756, dilution: 400×), rabbit anti-PMAT (Antikörper-online, Aachen, Germany, code ABIN754948, dilution: 1000×), rabbit-anti-OCT1/2 (Antikörper-online, code ABIN754948, dilution: 2000×) or rabbit anti-LDHA (Antikörper-online, code ABIN406429, dilution: 3000×) antibody followed by incubation with a secondary peroxidase linked anti-rabbit (Cell Signaling, code 7074, dilution: 1000×), or anti-goat (Santa Cruz Biotechnology, sc-2020, dilution: 5000×) antibody. For analysis of β-actin production, membranes were stripped, blocked by 2.5% (wt/vol.) BSA and incubated with mouse anti-β-actin antibody (Sigma-Aldrich, St Louis, MO, codeA5441, dilution: 20000×) followed by peroxidase-linked anti-mouse antibody (Sigma-Aldrich, USA; code A9044, dilution: 60000×). Protein production was visualized by luminol-enhanced chemiluminescence (ECL plus; GE Healthcare, Munich, Germany) and digitalised with Chemi-Doc XRS System (Bio-Rad Laboratories, Munich, Germany).

### The syngeneic orthotopic carcinoma model

Male C57BL/6J mice were purchased from The Jackson Laboratory (Bar Harbor, ME) and bred in our local animal facility. As published previously, laparotomy was performed on anesthetized mice (1.2–2.5% isoflurane), 2.5 × 10^5^ carcinoma cells were injected into the pancreas head, and the abdominal cavity was closed by sutures [20]. For pain relief 5 mg/kg carprofen (Pfizer GmbH, Berlin, Germany) was injected (sc) before surgery and 800 mg/L metamizol (Ratiopharm GmbH, Ulm, Germany) was added to the drinking water until euthanasia of the mice. Distinct mouse cohorts were either sham treated with an appropriate vehicle (PBS, ip), ip injected with gemcitabine (50 mg/kg) twice a week, ip injected with metformin (250 mg/kg metformin daily from day 8 to day 15; 125 mg/kg metformin daily from day 16 to day 29), or ip injected with a corresponding dose of gemcitabine plus metformin over a period of three weeks (Figure 1). For isolating the tumors animals were anesthetized with 90 mg/kg ketamine (bela-pharm, Vechta, Germany) and 7 mg/kg xylazine (Bayer Health Care, Leverkusen, Germany). All experiments were executed in accordance with the EU-directive 2010/63/EU and approved by the Landesamt für Landwirtschaft, Lebensmittelsicherheit und Fischerei Mecklenburg-Vorpommern.

### Analysis of the blood and tissue

The blood glucose concentrations were measured with the blood glucose meter Contour (Bayer Vital, Leverkusen, Germany) on day 14 and day 29 of the experimental schema in Figure 1A (1.5 hours after metformin injection). The concentration of leucocytes in the blood was determined on day 29 with the automated hematology analyzer Sysmex KX 21 (Sysmex Cooperation, Kobe, Japan). The tissue was sampled on day 29 and processed as described previously [19]. The histology of the tumors was evaluated on haematoxylin and eosin (H/E) stained paraffin tissue sections. For visualizing the pH in the carcinoma, we invented a new method: In a CM1850 cryostate (Leica Mikrosysteme Vertrieb GmbH, Wetzlar, Germany) 10 μm sections were cut and placed on white writing paper, which was freshly soaked in 1% bromothymolblue solution (SCS GmbH, Bonn, Germany). Prior to soaking the paper in the indicator solution, the color of the solution was adjusted with 1 M NaOH until a green color was observed. Photos were taken within 15 seconds of placing the tissue section onto the soaked paper. In order to measure the pH, the liquid within the central cyst of the tumor was removed with a syringe and the remaining tissue was minced. Solid particles in liquid and minced tissue were removed by centrifugation and the pH in the supernatant was measured by a RapidLab Analyzer (Siemens Healthcare GmbH, Erlangen, Germany).

### Evaluation of cell death, proliferation and protein expression *in vivo*

Cell death was analyzed on paraffin tissue sections using the ApopTagPlus Peroxidase *in situ* detection kit (Millipore). Cell proliferation and transport protein expression was evaluated by immunohistochemistry using mouse anti-BrdU (Dako, Hamburg, Germany, clone Bu20a, dilution: 50×), goat anti-CNT1 (Santa Cruz Biotechnology, code sc48457, dilution: 200×), rabbit anti-CNT3 (Santa Cruz Biotechnology, code sc134529, dilution: 200×), rabbit anti-ENT1 (Abcam, code ab135756, dilution: 500×), rabbit anti-PMAT (Antikörper-online, code ABIN754948, dilution: 300×), rabbit-anti-OCT1/2 (Antikörper-online, code ABIN754948, dilution: 800×). The Universal LSAB+ Kit/HRP (Dako) was used as secondary antibody.

### Data presentation and statistics

Data presentation and statistics were performed as described previously [19, 20]. Box plots indicate the median, the 25th and 75th percentiles in the form of a box, and the 5th and 95th percentiles as whiskers. The significance of differences was evaluated using a Mann-Whitney rank-sum test followed by the correction for the accumulation of the α error by considering the number of meaningful comparisons (Bonferroni correction). Differences with *P* ≤ 0.05, divided by the number of meaningful comparisons, were considered to be significant. Differences with *P* ≤ 0.08, divided by the number of meaningful comparisons, were considered to indicate a tendency.

## SUPPLEMENTARY MATERIALS FIGURE


