# Supplementary material for: Intratumoral heterogeneity of the therapeutical response to gemcitabine and metformin
Source: Oncotarget. 2016 Jul 28;7(35):56395–407. doi: 10.18632/oncotarget.10892 (PMC5302922; doi:10.18632/oncotarget.10892)
Supplement: Supplementary file 1 [file oncotarget-07-56395-s001.pdf]

Intratumoral heterogeneity of the therapeutical response to gemcitabine and metformin

Supplementary Materials

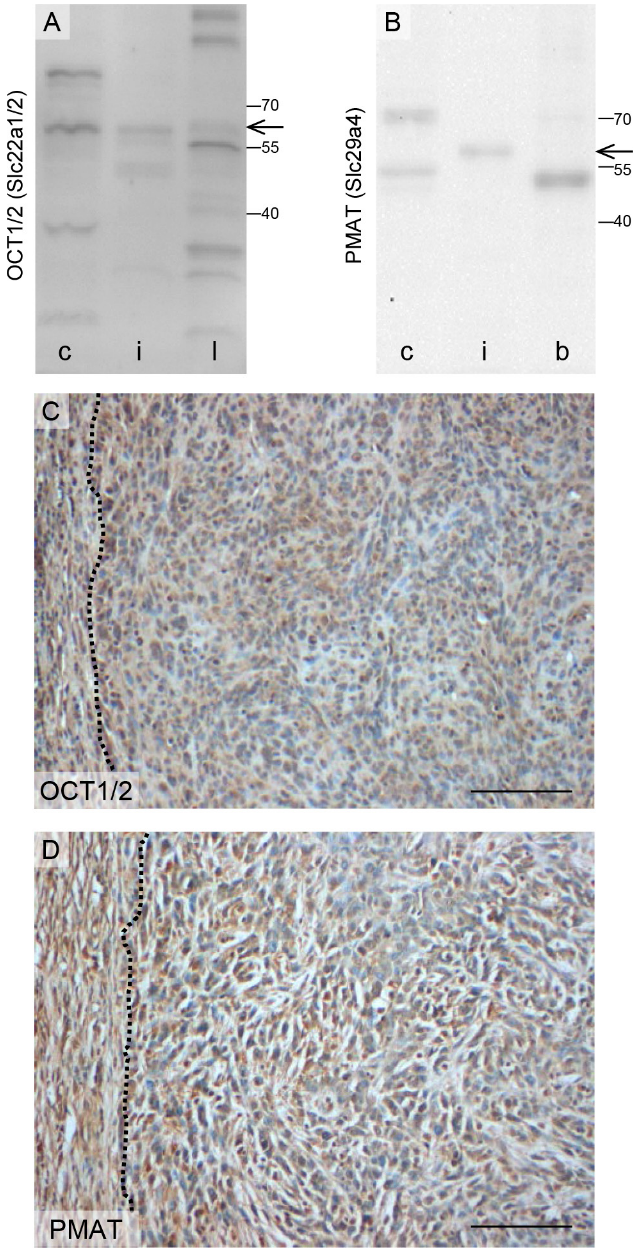

Supplementary Figure S1: Expression of proteins involved in metformin transport. Analysis of OCT1/2 (**A**), and PMAT (**B**) expression in 6606PDA cells (**c**), intestine (**i**), liver (**l**) and brain (**b**) by Western Blotting (the arrows point at OCT1/2 or PMAT with a theoretical molecular weight of 62kD and 58 kDa, respectively). Evaluation of OCT1/2 (**C**) and PMAT (**D**) expression in carcinomas by immunohistochemistry (the broken lines indicate the border between the carcinoma and the desmoplastic reaction). Bar = 100 µm.
